# Supplementary material for: A New Gold(III) Complex, TGS 703, Shows Potent Anti-Inflammatory Activity in Colitis via the Enzymatic and Non-Enzymatic Antioxidant System—An In Vitro, In Silico, and In Vivo Study
Source: Int J Mol Sci. 2023 Apr 10;24(8):7025. doi: 10.3390/ijms24087025 (PMC10138903; doi:10.3390/ijms24087025)
Supplement: Supplementary file 1 [file ijms-24-07025-s001.zip › ijms-2321347-supplementary.pdf]

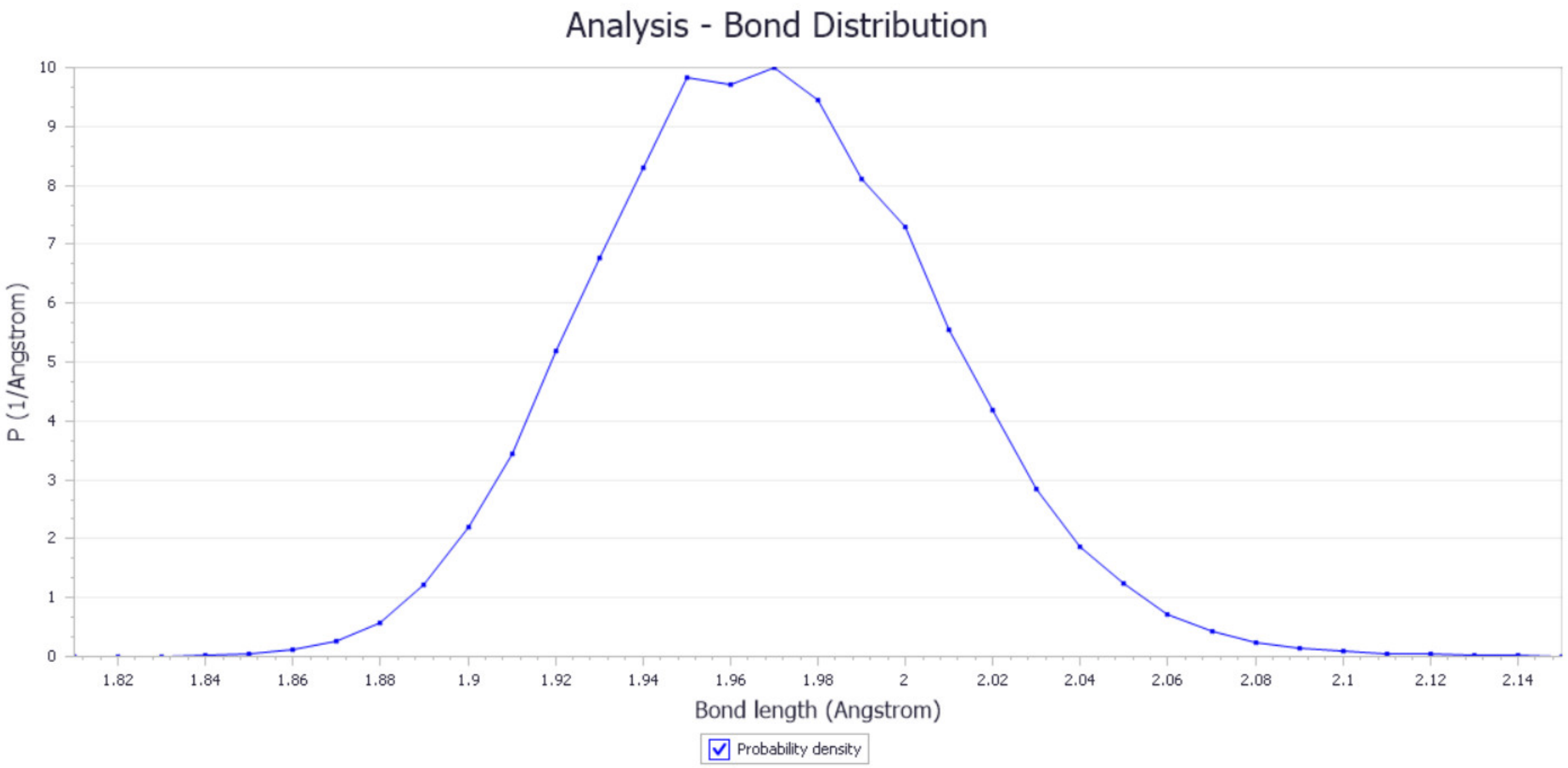

Figure S1. Bond distribution in $Au(CN)_4^-$ during the molecular dynamics simulation.

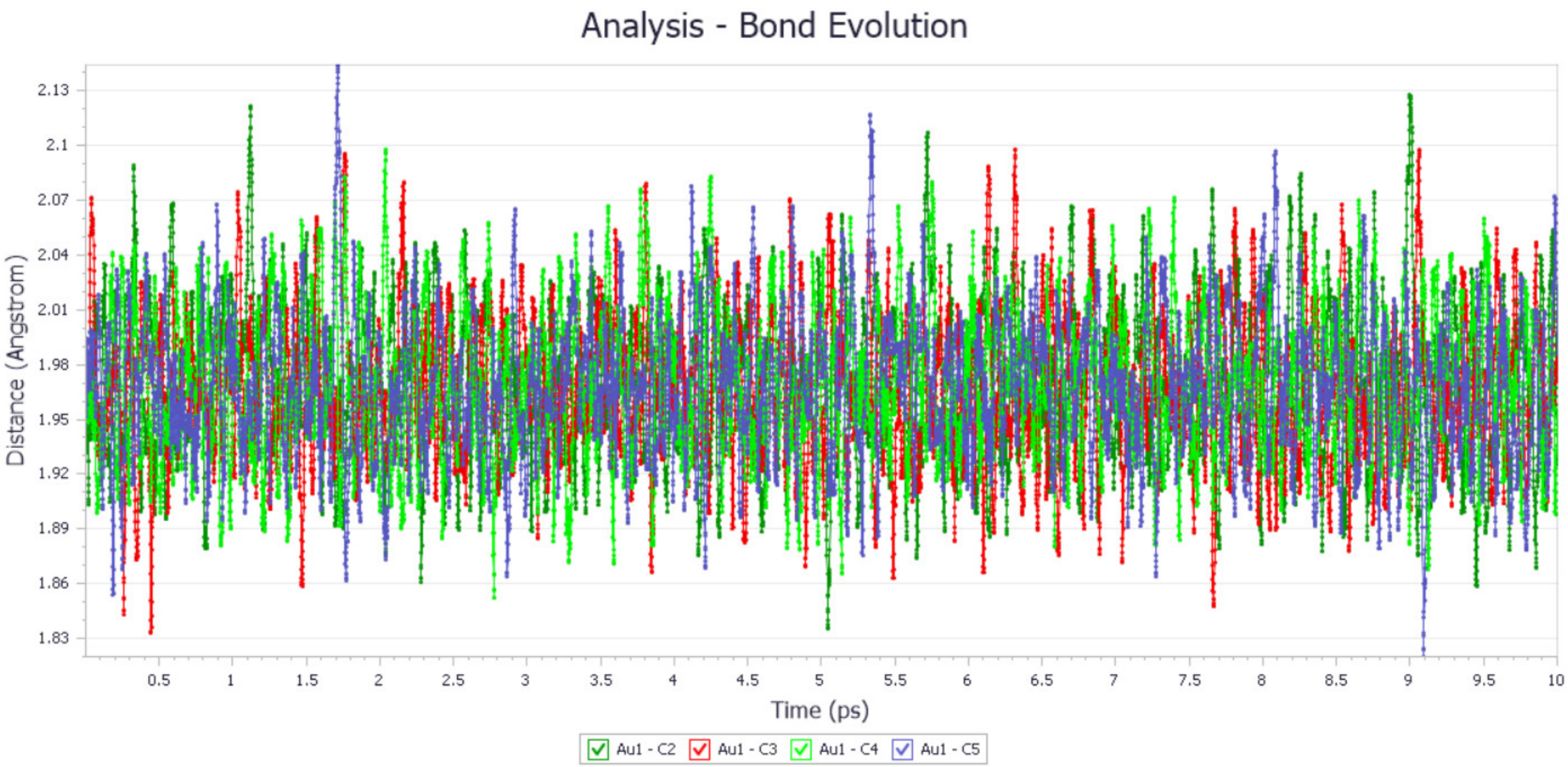

Figure S2. Bond evolution in $Au(CN)_4^-$ during the molecular dynamics simulation.

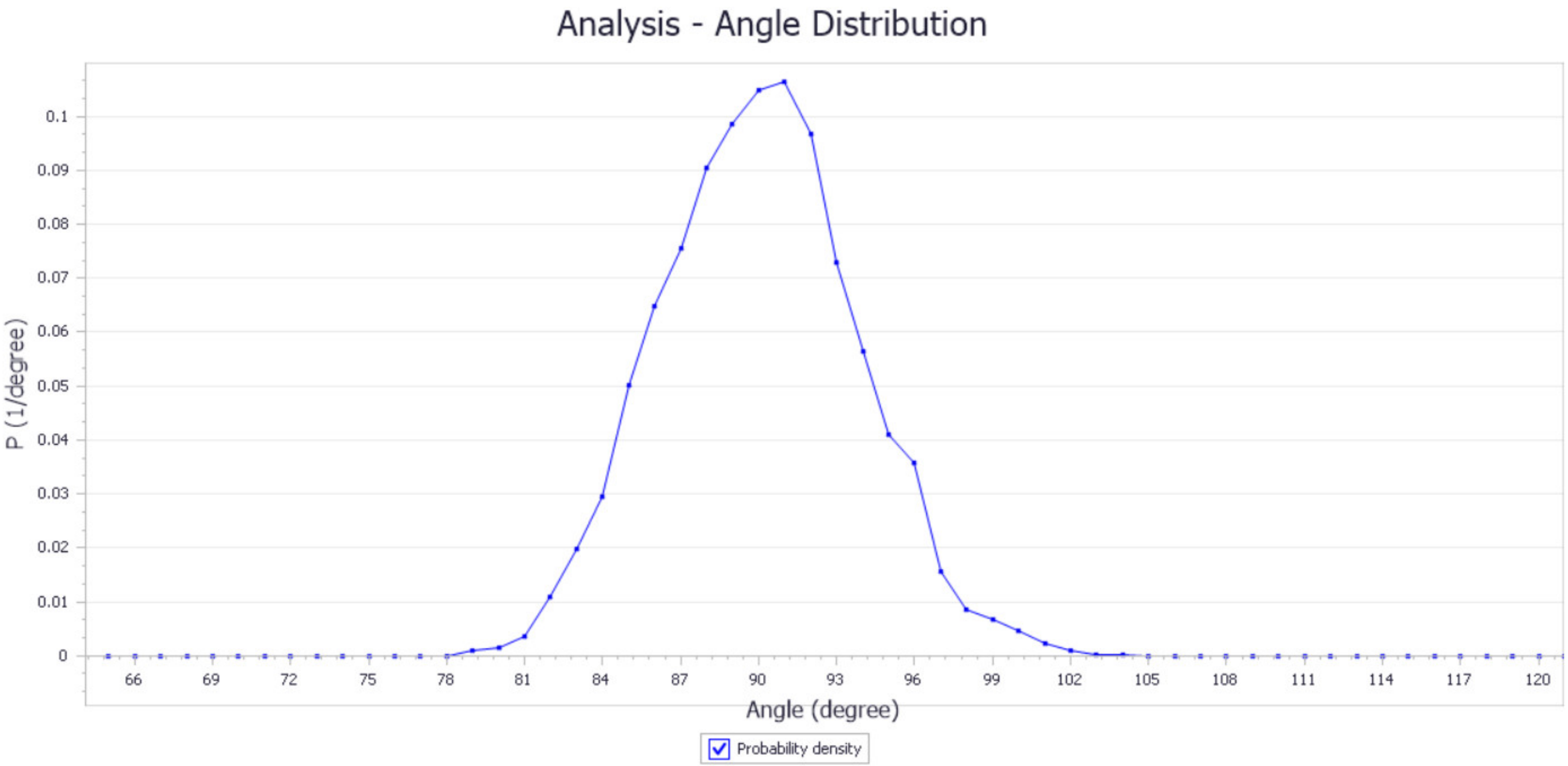

Figure S3. Angle distribution in $Au(CN)_4^-$ during the molecular dynamics simulation.

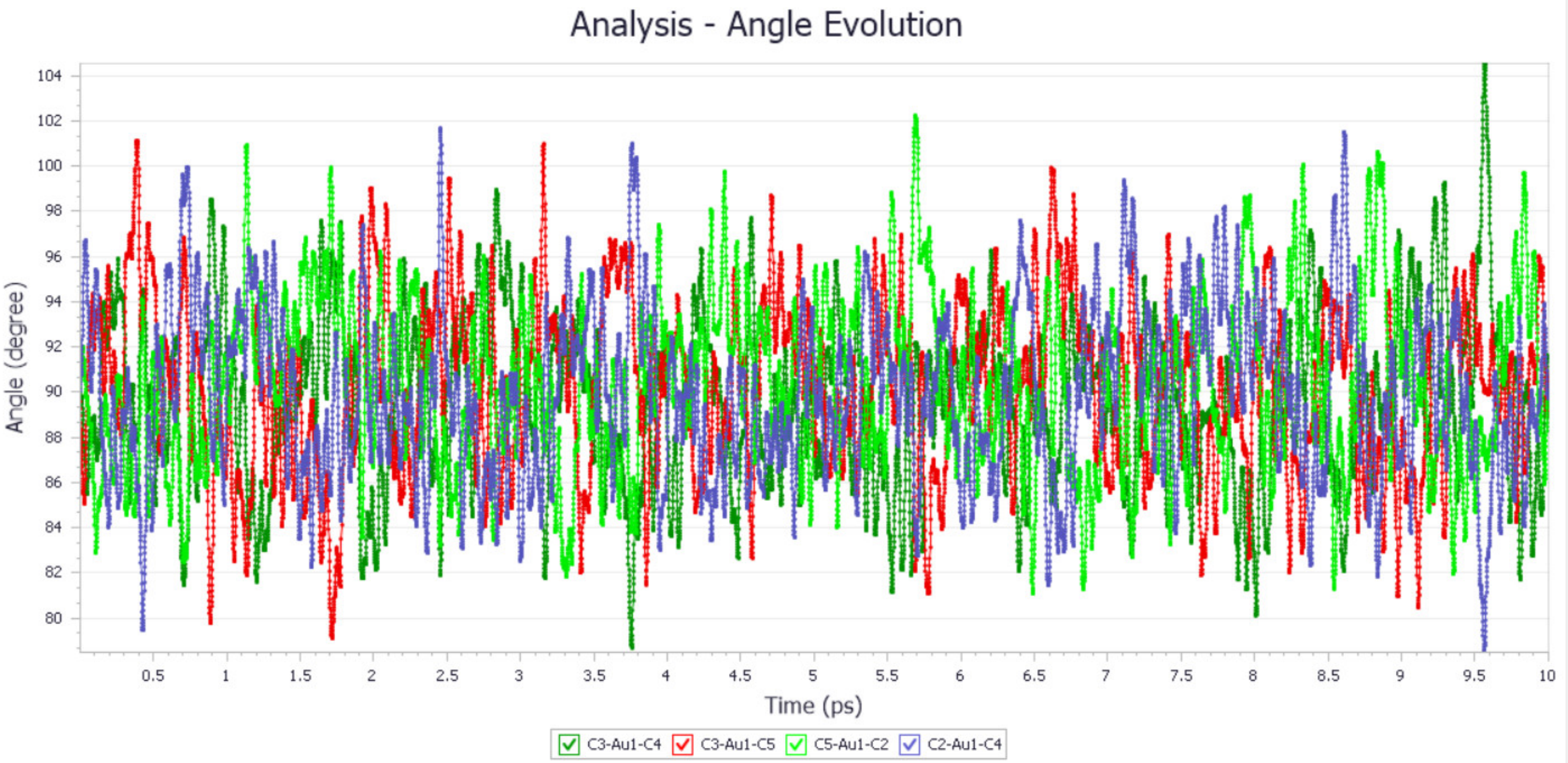

Figure S4. Angle evolution in $Au(CN)_4^-$ during the molecular dynamics simulation.

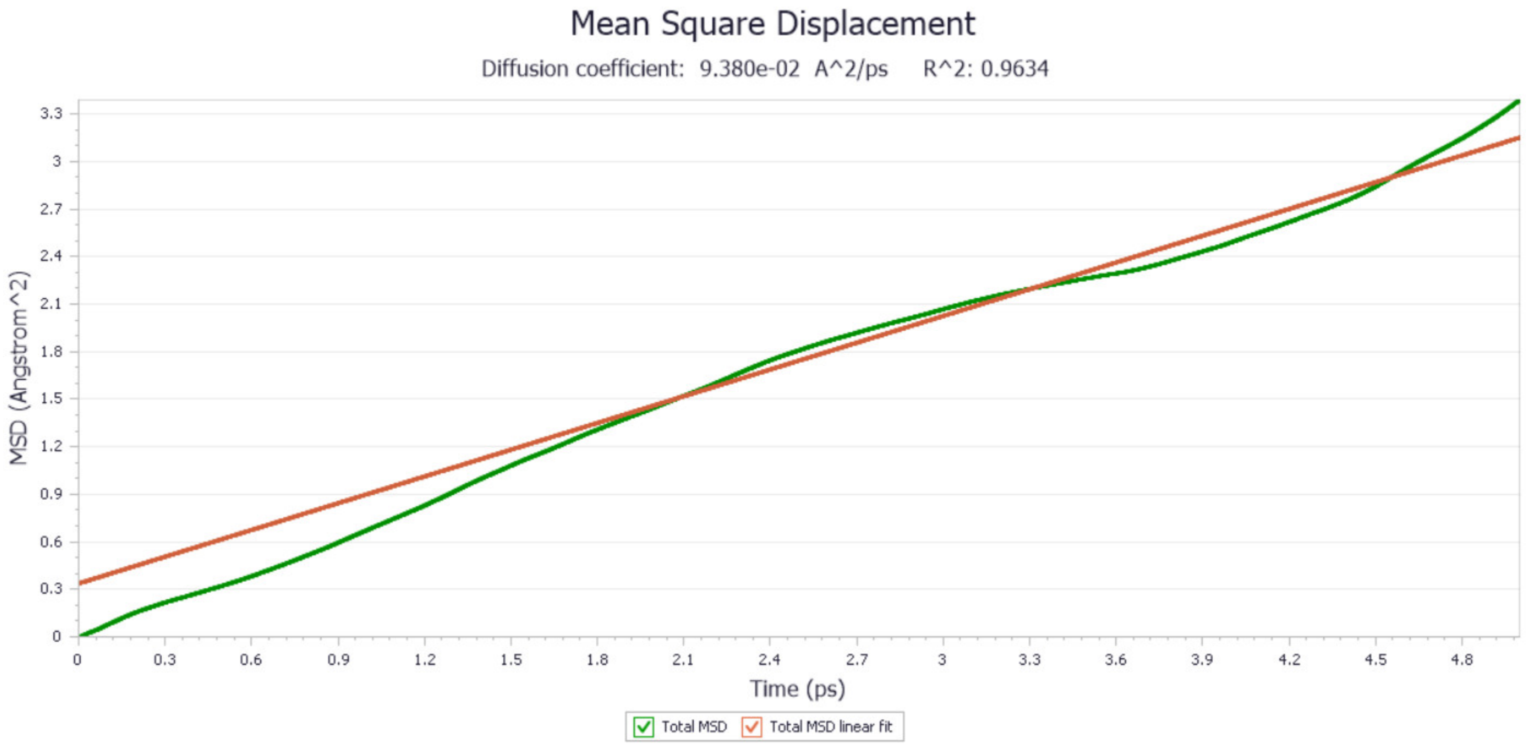

Figure S5. Mean square displacement of $Au(CN)_4^-$ during the molecular dynamics simulation.
